# Validation of collaborative cyberspace virtual reality oculometry enhanced with near real-time spatial audio

**DOI:** 10.1038/s41598-023-37267-x

**Published:** 2023-06-21

**Authors:** Peter M. Maloca, Javier Zarranz-Ventura, Philippe Valmaggia, Balázs Faludi, Marek Zelechowski, Adnan Tufail, Norbert Z. Zentai, Hendrik P. N. Scholl, Philippe C. Cattin

**Affiliations:** 1 Institute of Molecular and Clinical Ophthalmology Basel (IOB), 4031 Basel, Switzerland; 2 Department of Ophthalmology, University Hospital Basel, 4031 Basel, Switzerland; 3 Moorfields Eye Hospital NHS Foundation Trust, London, EC1V 2PD UK; 4 Hospital Clínic of Barcelona, University of Barcelona, 08036 Barcelona, Spain; 5 Centre for Medical Image Analysis & Navigation, University of Basel, 4123 Allschwil-Basel, Switzerland

**Keywords:** Medical research, Translational research

## Abstract

Currently, most medical image data, such as optical coherence tomography (OCT) images, are displayed in two dimensions on a computer screen. Advances in computer information technology have contributed to the growing storage of these data in electronic form. However, the data are usually processed only locally on site. To overcome such hurdles, a cyberspace virtual reality (csVR) application was validated, in which interactive OCT data were presented simultaneously to geographically distant sites (Lucerne, London, and Barcelona) where three graders independently measured the ocular csVR OCT diameters. A total of 109 objects were measured, each three times, resulting in a total of 327 csVR measurements. A minor mean absolute difference of 5.3 µm was found among the 3 measurements of an object (standard deviation 4.2 µm, coefficient of variation 0.3% with respect to the mean object size). Despite the 5 h of online work, csVR was well tolerated and safe. Digital high-resolution OCT data can be remotely and collaboratively processed in csVR. With csVR, measurements and actions enhanced with spatial audio communication can be made consistently in near real time, even if the users are situated geographically far apart. The proposed visuo-auditory framework has the potential to further boost the convenience of digital medicine toward csVR precision and collaborative medicine.

## Introduction

Effective and reliable communication of medical data is essential for the integration of digital content in medicine. Digital medicine^1^ therefore uses software and algorithmically managed components to assess human health and store and distribute acquired data. In medicine, the Picture Archiving and Communication System (PACS)^2^ is an example of a picture archiving and communication system based on digital computers and networks. Although central PACS technology has advanced, the main operational problems of implementing a PACS over an internal local area network (LAN) and, consequently, the associated maintenance and troubleshooting costs remain^3^.

The concept of virtual reality (VR) was conceived in the 1960s as a possible gateway through which a user can experience synthetic content in a realistic way^4^. Since that time, sophisticated VR devices have been developed that offer stereoscopic immersion into a computer-generated digital environment using head-mounted displays (HMDs)^5^. Such VR systems have been widely used in different medical fields, such as for therapeutic applications^6–8^, pre-surgery simulation^9^ and planning^10,11^, and medical education^12,13^. Despite the great success, some limitations and challenges became apparent, such as the implementation of VR technology in a user-friendly way and the high technological requirements, especially those for the graphic representation of complex content, resulting in relatively high costs for a mainly isolated single user-adopted VR experience^14^.

In recent years, optical coherence tomography (OCT) as a noninvasive cross-sectional imaging technology in human tissue has become the gold standard in ophthalmic imaging^15,16^. Traditionally, OCT images are displayed in two dimensions on a computer screen, resulting in spatial and temporal restrictions to sharing precious information. Today, OCT image display capabilities have been enhanced using VR^17^. Three-dimensional OCT models created with VR were successfully perceived and used as an appropriate digital interface to enhance medical image display^18,19^ in an interactive way in lay and medical expert settings^17,20,21^. These qualitative studies are very important in promoting the transition of VR into medical practice. However, we posit that only precise navigation, which is also quantifiable and reproducible in VR, can increase trust and confidence in this digital medical technology. Therefore, on a macro level, our group previously showed with measurements of the human skull that VR is a suitable and valid technology to unite the digital world with physical world measurements^22^.

In this study, we aim to overcome some of the limitations mentioned above, such as the status of a solitary and isolated VR user situation or the lack of ocular VR, particularly retina and choroid quantification, and take VR to the next medical level. We performed repeated diameter measurements in the OCT micrometer range of a healthy human eye. We integrated an additional level of difficulty by having these measurements independently conducted by three experienced experts at different clinical sites geographically located far apart and connected only by our proposed cyber space virtual reality (csVR) application marked as Specto. In the definition of cyberspace, we adopt the recommendation of the European Court of Auditors, specified as “The intangible global environment in which online communication occurs between people, software, and services via computer networks and technological devices”^23^.

Here, we demonstrate rigorous advances in accessing and handling virtually tangible quantitative imaging data in cyberspace over a physically long distance. To this end, our study makes specific significant contributions. First, the repeated ocular navigation and measurements performed showed reproducible values, indicating that complex csVR experiments can be carried out reliably. Second, stable VR environments connected through cyberspace were successfully created, boosting collaborative work between geographically distant medical experts embedded and enhanced by near to real-time visual and auditory exchange. Third, the high degree of csVR coupling that allowed for direct interaction was positively perceived, and users did not encounter any significant side effects despite performing complex tasks for five hours.

Thus, the proposed csVR solution Specto can be the cornerstone for reliably promoting digital transformation in medicine and developing new concepts that are not realisable in the physical world. Further, csVR can be made accessible to less privileged individuals and enable entirely new health IT platforms and precise digital interventions to improve health outcomes.

## Results

**Figure 1:**
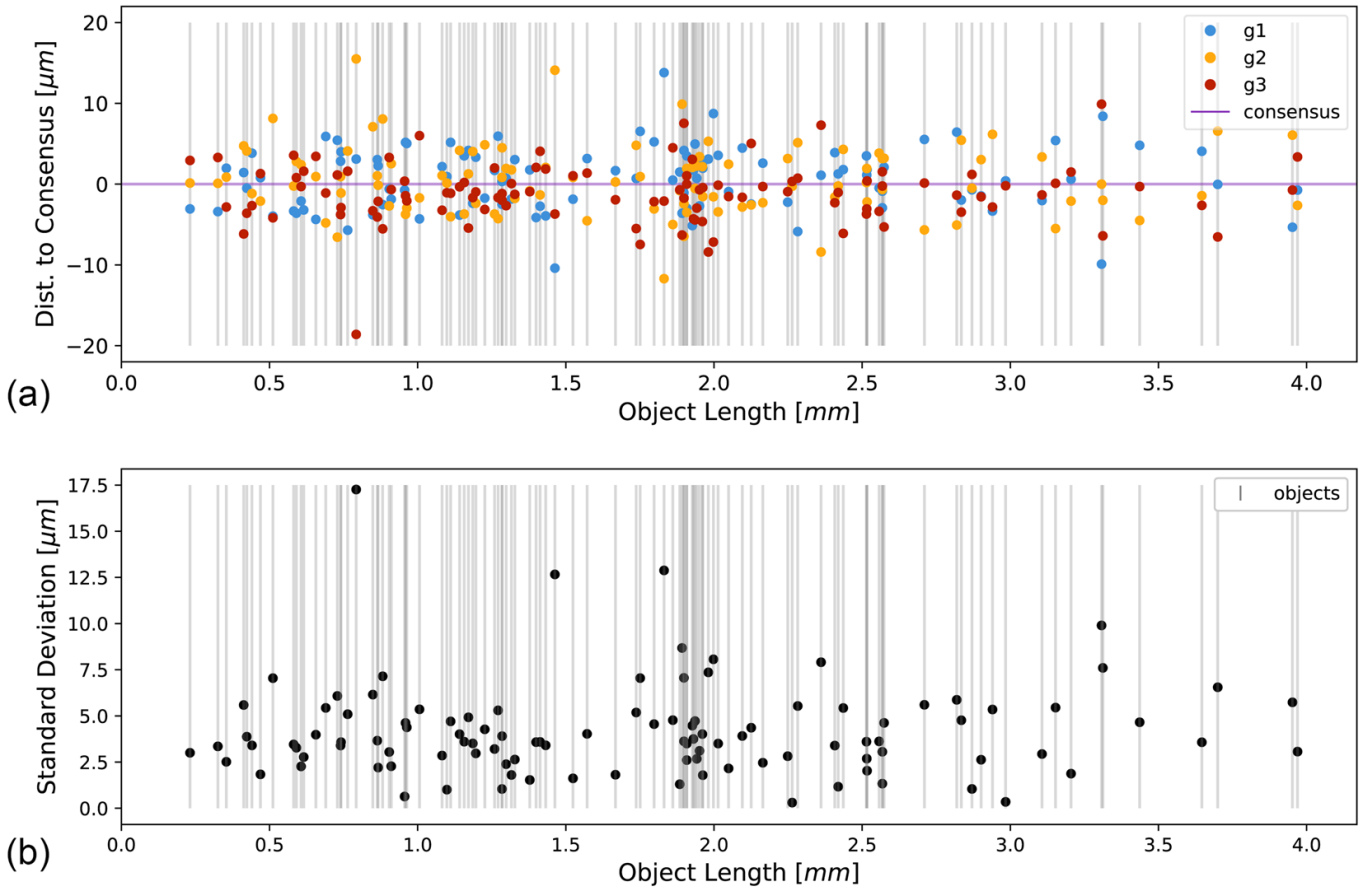
Visualisation of the 327 length measurements made in the csVR of 109 objects. The x-axis shows the object length in mm. Each grey horizontal bar indicates one object’s mean of the three length measurements that were made for this object, that is, the consensus. (**a**) The y-axis indicates the deviation from the consensus in μm for each of the three measurements. The blue, yellow, and red points visualize the individual measurements made by graders g1, g2, and g3, respectively. The horizontal violet line visualizes the consensus. (**b**) The y-axis indicates the standard deviation in μm calculated from the three length measurements for each of the 109 objects.

All 327 recorded length measurements are shown in the scatter plot in Fig. 1a. The recorded length measurements of the 109 objects ranged from 0.23 mm to 3.97 mm, with a mean of 1.73 mm and a median of 1.75 mm (Fig. 1a, Table 1). The absolute difference between two measurements of the same object ranged from 0 μm to 34.1 μm with a mean of 5.3 μm and a median of 4.5 μm. The standard deviations calculated from the three measurements for each of the 109 objects ranged from 0.3 μm to 17.2 μm with a mean of 4.2 μm and a median of 3.6 μm (Fig. 1b). The coefficient of variation (CoV) calculated for each of the 109 objects ranged from 0.012 to 2.18%, with a mean of 0.34% and a median of 0.24%.

**Table 1:** Distance matrix of mean absolute difference between length measurements of graders g1, g2, g3, and the consensus in μm.

	g2	g3	Consensus
g1	5.7	5.1	3.2
g2		5.1	3.2
g3			2.8

**Figure 2:**
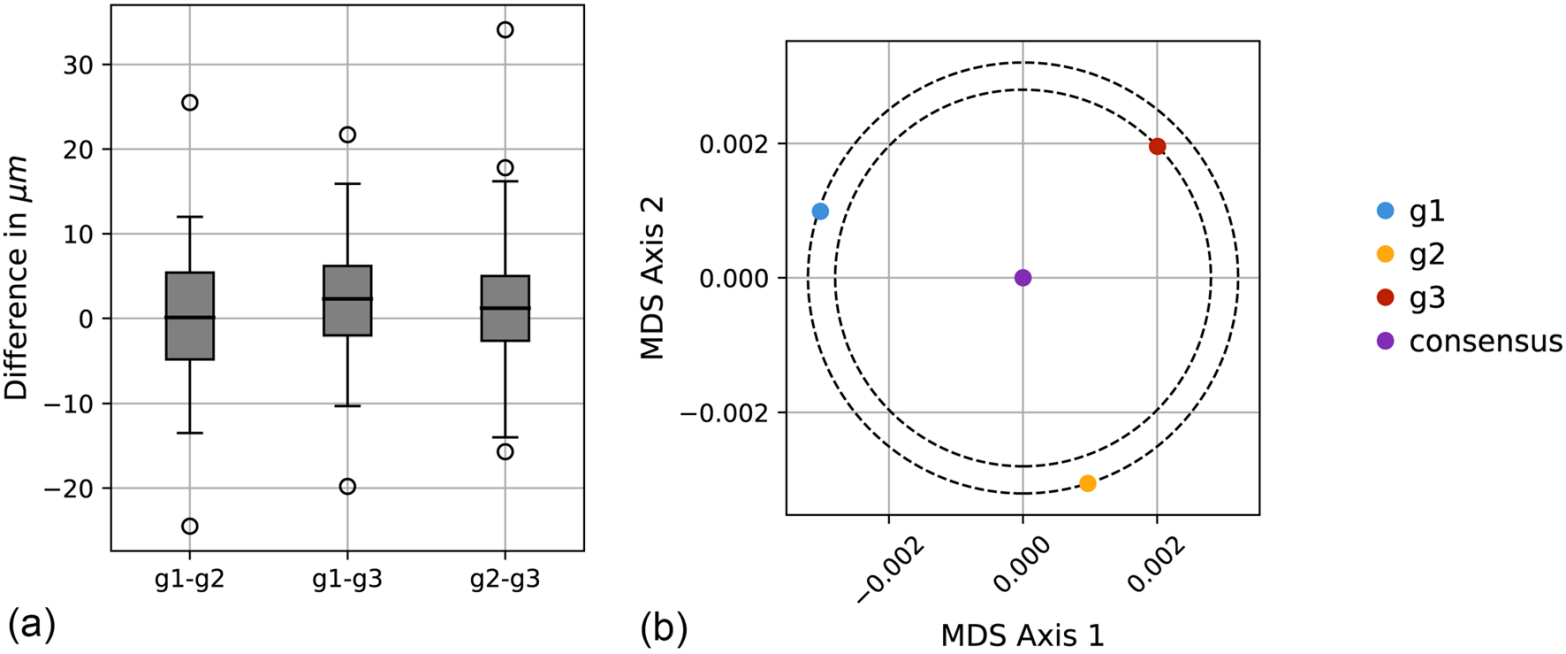
Visualisation of differences in annotated lengths among the three graders, g1, g2, and g3. (**a**) Boxplots of length differences between g1 and g2 (left), g1 and g3 (middle), and g2 and g3 (right). Boxes extend from the end of the first quartile to the end of the third quartile, with a black line at the median. Whiskers indicate the range of the data, and white points are past the end of the whiskers. (**b**) Multidimensional scaling (MDS) plot of g1, g2, g3, and the consensus. MDS converts distances among data points into positions in a two-dimensional coordinate system while preserving these distances as well as possible. Axes are arbitrary and usually do not have an intuitive interpretation. The MDS plot was centered at the consensus (violet dot). g1 and g2 are approximately equally far from the consensus, as indicated by the outer dashed circle in black. g3 is a bit closer to the consensus, as indicated by the inner dashed circle in black.

Differences in length measurements of the 109 objects are shown in the boxplots in Fig. 2a. The boxplot on the left shows the differences between grader g1 and g2. The boxplots in the middle and on the right show the differences between g1 and g3 and between g2 and g3, respectively. A distance matrix with the mean absolute difference of the length measurements among the graders and the consensus is shown in Table 1. A multidimensional scaling plot based on the differences in Table 1 is shown in Fig. 2b. The ANOVA analysis detected a significant effect of the independent variables “grader” (p = 0.02) and “object ID” (p < 2 × 10^–16^) on the dependent variable “object length” (Table 2). The independent variable “line color” did not have a significant effect (Table 2).

**Table 2:** ANOVA table.

	Sum Sq	Df	F-value	p-value	Significance
Grader	0.00015	2	3.97	0.02	*
Object ID	251.99273	108	126,461.91	< 2 × 10^–16^	***
Line Color	0.00030	15	1.07	0.39	
Residuals	0.00364	197			

Stars indicate the significance of p-values: *** < 0.001 ≤ ** < 0.01 ≤ * < 0.05.

All measured values for statistical analysis and for generating the figures and tables are summarised in Supplementary File S1.

### Subjects and data connectivity

All participants in the study were male, with a mean age of 41.3 years (ranging from 28 to 55 years). All were ophthalmologists with a mean medical work experience of 15.33 years (range from 4 to 26 years), mean ophthalmological work experience of 14.33 years (range from 4 to 23 years), and mean VR experience of 4.67 years (range from 2 to 7 years). The internet mean upload speed was 595 megabits per second (range from 36 to 900 megabits per second), mean download speed was 427 megabits per second (range from 0.86 to 650 megabits per second), mean unloaded latency 115 ms (range from 2 to 340 ms), and loaded latency was 213 ms (range from 3 to 628 ms).

### Qualitative VR software and hardware analysis

**Figure 3:**
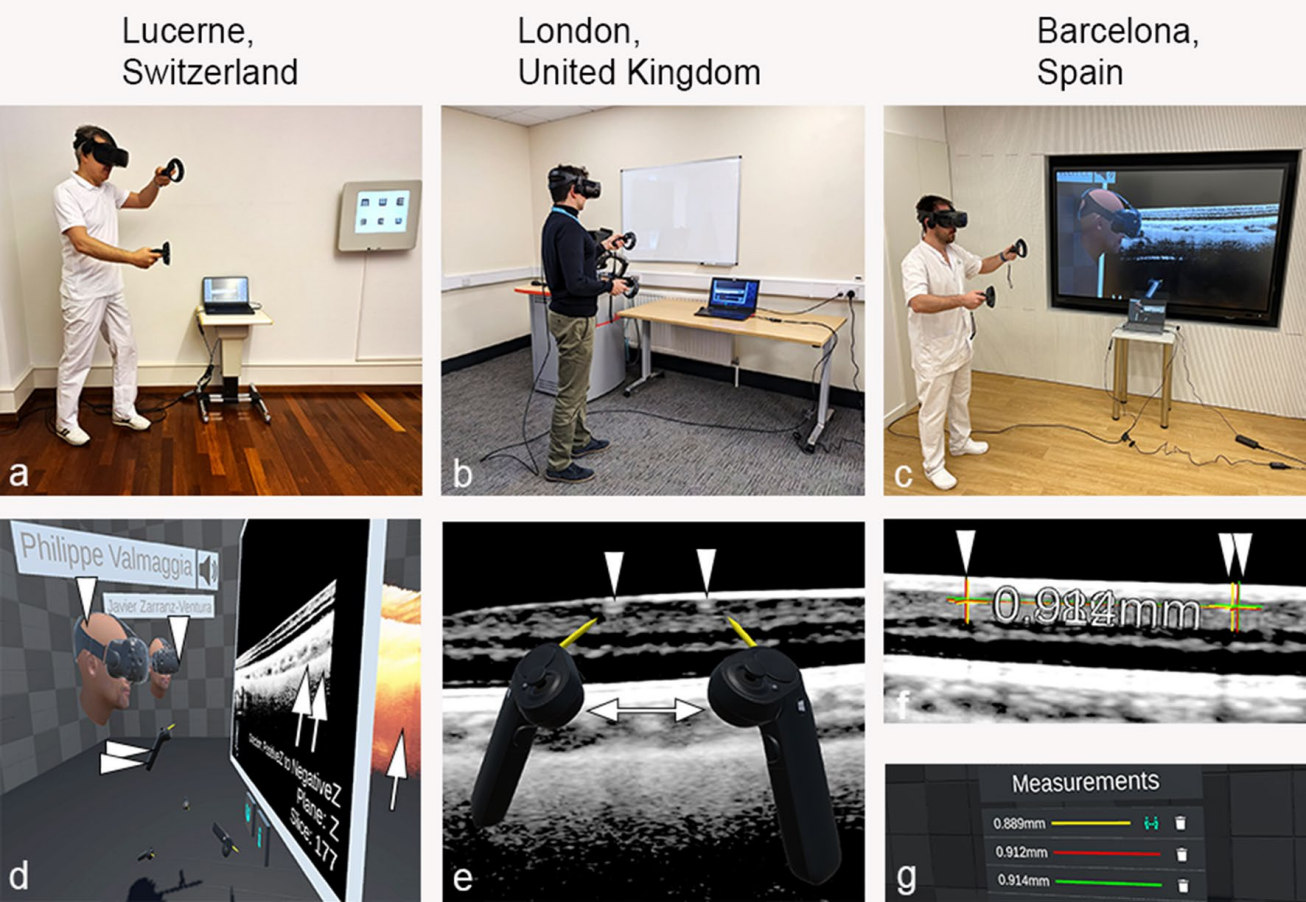
Overview of collaborative oculometry in cyberspace virtual reality (VR) for measuring digital medical data. (**a**–**c**) Three graders were physically located in three geographically distant places. Using the proposed cyberspace VR software, graders were enabled to view and precisely navigate in an interactive and virtual environment. They entered the cyberspace play arena with their physical devices (**d**). In the image, two graders are illustrated as medical avatar cybernauts (PV and JZ, single white arrowheads) holding the two VR handles per person (double arrow heads). An orange-yellow coloured portion of the volume rendered OCT object is depicted (single arrow), the cross section of which shows the B-scan number 177 (double arrows). The third user (PMM) took this image with a virtual digital camera and is therefore not shown. (**e**) In alternating sequence, a grader showed which diameters of the retina or choroid were to be measured and indicated the measuring landmarks with the yellow live tips of his csVR handle (double arrow). Two hyperreflective dots (single arrowheads) that correspond to retina vessels in the cross-section are pinpointed. However, no permanent marking was placed to avoid influencing the graders. (**f**) The measurement lines and the measurement lengths were immediately hidden and displayed only after the measurements were completed by all graders (**g**). Subsequent diameters were measured in the same way. (**f**) shows that the left position (single arrowhead) was marked very similarly by the three graders, whereas the right position in this example showed some variation (double arrowheads).

In terms of personal assessment, all graders reported that the proposed VR application was technically well suited for a validation study. As depicted in Fig. 3g, the choice of color for the measuring ruler was determined at random by the software and changed after each diameter measurement. It turned out that lighter colors, such as red, green, or blue, were preferred to violet or brown by the graders. In addition, all graders stated that the instant spatial audio function was exceptionally helpful in localizing the other graders in the VR space and in giving direct instructions to each other. The speed of the VR manipulation was perceived as being close to reality manipulations by all graders. The OCT image display in the virtual space seemed comparable to the conventional computer display on a computer screen but more intuitive. The greatest strengths were the intuitive interaction with OCT data, scaling options, quick switch from 2 to 3D data representation, and clear communication with other users. No one from the grader considered that it was an unnecessary approach and was able to complete the tasks even after five hours of collaborative work.

However, two graders reported short audio losses during the study. The sharpness of the displayed OCT images was acceptable, but the graders requested a larger field of view. The major limitations were the advanced technical needs (high-end computers and tethered cables). Another drawback was the relatively time-consuming start-up procedure, with time loss for starting the computer, launching the Windows Mixed Reality software, and the initiation of the VR software. Further, the exact positioning of the landmarks could, in part, be difficult due to hand tremor.

A full list of the comments by the graders is provided in Supplementary File S2.

### Simulator Sickness Questionnaire and software evaluation

The analysis of the completed Simulator Sickness Questionnaire (SSQ) showed values of 0, 3.74, and 7.48, with a mean value of 3.74.

## Discussion

Through the use of sophisticated computer technology, VR has enabled the perception of digital three-dimensional medical data, in which objects appear to have a sense of spatial representation^17^. Similar solutions have promoted VR to be implemented in medicine^24^ and in preparing patients^25^, nurses^26^, doctors and surgeons^27^ for real-life practice and procedures.

However, despite these great successes, current VR applications have mainly been adopted for qualitative investigations, and only a few reports exist on spatial VR measurements and on their reproducibility^22,28^. To take VR to the next level, quantitative research is needed to generate objective data that can be reported through numbers and statistics. Thus, based on the repeated diameter measurements of all the objects measured in this study, we found a minor standard variation of 4.2 µm. This csVR-derived value, which corresponds to a slight deviation of only 0.3% of the mean object size, is lower than the axial resolution of a standard OCT device^29^ and is in good agreement with other comparable studies conducted in a local user setting^22,28^. The observed variability among the three graders in their length measurements was relatively uniform (Table 1, Fig. 2b), even though there were small systematic differences that were significant (Table 2, Fig. 2a). The low variation in measurements allows for unbiased reproducibility of a csVR solution by other groups, which, in turn, will increase confidence in VR. This is more important because future advances will allow for digital navigation decisions in a surgical VR realm, and potentially from geographically distant locations. For tele-pre-surgery training, we envision that such a csVR setup can enhance the procedure for robotic-assisted intravitreal injections^30,31^, cataract surgery^9,32^, or vitreoretional surgery^33–35^.

We wanted to address another weakness of current VR by extending the existing single-user solution^17^ into a cyberspace-based merged workspace. To do this, the previous VR software was further developed into a collaborative VR space where users could independently perform measurements on the same model from a distance rather than in one physical space in one location. Such collaborative work has been reported previously for medical education^36^ and data exploration^37^, but no data exist on cyberspace diameter measurements, particularly on their reproducibility and validation. The questionnaires showed that the graders experienced a close and instant coupling to the cyberspace VR solution with no detectable time lag, even though the range of internet speeds was relatively wide. They did not experience any noticeable delays or frame drop-outs, allowing for continuous collaboration without any major issues during the study. In addition to the visual representation, the graders also appreciated the direct possibility of being able to discuss the situation via direct VR spatial audio and coordinate the measurements in an alternating sequence. They reported that it was nevertheless helpful that a consensus was reached prior to the start of the study on which structures should actually be measured. The ability to create hidden measurements and, after each grader finished the individual annotation, disclosing them as an overlay was greatly appreciated. This allowed to discuss similarities and differences in the measurements live in cyberspace. Rapid medical consensus finding hence becomes feasible in three dimensional data. The consistency of the csVR performance was all the more remarkable because the measurements were carried out simultaneously in geographically distant locations and on different computers. The consistency and precision of the measurements as well as the option of live interaction makes csVR discussions an excellent option for a live consensus finding, where difficult clinical cases can be discussed qualitatively and quantitatively.

### Quality of experience for cyberspace virtual reality

To adopt VR for digital medicine, various preconditions must be satisfied. Prior experience with VR systems may not protect a user from potential nausea if the immersion is technically poorly implemented. Thus, the quality aspects are of utmost relevance, not only because they are related to the execution of tasks but also for the comfort and well-being of the VR operator. Therefore, in this study, we also assessed the quality of the cyberspace VR experience. In accordance with the literature^38,39^, the obtained mean SSQ value of 3.74 expressed negligible csVR symptoms. Thus, the SSQ survey showed good acceptance and tolerance, as previously reported for adults^17^ and children^21^ but in a local VR setting. Obviously, the current number of users was, of course, too small to allow for drawing concrete statistical conclusions.

### Limits of csVR

The number of study participants in this study was relatively small; thus, further studies with larger numbers are recommended. We consider a group of three people ideal for collaborative work so that the scene remains organised and comprehensible. Nevertheless, future studies should examine how the procedure can be conducted with a large number of graders. All users used the identical HMD brand. In future studies, the performance of other HMD brands or additional immersive technologies, such as augmented or mixed reality, can be compared because differences have been reported^40^. Only diameter measurements were carried out in this study; thus, future investigations could include measurements of angles, areas, or volumes. Nevertheless, this study is of central importance, because a diameter is the fundamental source of all other measurement objects, and we demonstrated that these measurements were extraordinarily consistent. A limit may be that the number of graders was rather small and that the graders had some prior experience with virtual reality. However, only one grader (PMM) was familiar with the proposed VR application. The other graders (PV, JZV) entered the VR application without prior knowledge and experience, so that a selection bias must be subordinate in the choice of the graders. In addition, the assessment of motion sickness was not the primary goal.

Another possible limitation of the measuring accuracy could be the brief display of the measuring marks with the yellow handle tip, without being permanently marked, and hiding the measuring lines from other graders until the end of the measurement. Thus, the starting and ending points of measurement were at the discretion of individual graders. Despite this ambiguity, the measurements were robust. An advancement could be a marking tool that can snap a measurement on a particular structure, depending on its image contrast intensity. The computer operating system used was Microsoft Windows® (Microsoft Corporation, Redmond, US). Whether the csVR would deliver equivalent results on Apple IOS, Linux, or other operating systems remains to be examined. A comparison was also not yet possible, as the VR device used was primarily compatible with only the latest version of Windows 10. A further limitation is that the digital VR measurements have not yet been compared with measurements made with traditional imaging software. This extension is the subject of upcoming studies. Further limitations of VR are the relatively expensive hardware, limited screen resolution and reduced visual field of view, insufficient and heavy HMDs, and the need for tethered computer communication for fast and continuous data transfer. Although in the current csVR application, visual interactions were enhanced by real-time spatial audio, there is still a lack of haptic feedback. In addition to speech- or image-based interactions, haptic feedback would allow users to experience the virtual environment and the virtual model through touch. This would be very helpful, especially for future tele-surgical interventions. Arguably, the use of the SSQ can be discussed. Nevertheless, we consider this tool helpful because the graders had to perform a high-precision work in VR for more than five hours. The result of the current SSQ was positive. In addition, the identical SSQ test has been used in the past with other physicians, laymen and even children. So, we can also show a good tolerance compared to previous time points.^17,21^.

The National Aeronautics and Space Administration (NASA) has successfully installed a SPECTRALIS imaging platform on the International Space Station (ISS) since 2013, to better investigate the effects of microgravity on vision. Thus, further considerations of future digital medicine and virtual reality based OCT image analysis also need to address the impact of space missions on eyes^41^ that could compromise an intended flight. Spaceflight-associated neuro-ocular syndrome represents a complex syndrome of findings and symptoms identified in astronauts who have completed long-duration missions in microgravity^42^. Possible complications that can be detected by OCT on the space station include disc edema, choroidal folds, cotton wool spots, nerve fiber layer thickening on OCT, globe flattening, and hyperopic shift^43,44^. These pathologies can remain even after a mission and cause long-term damage. In this context, it is important to explore the relationship between the eye and the cerebral fluid compartments with OCT directly in space. Potentially, the proposed csVR could also be a digital communications tool for even more distant researchers and enable a direct visual discussion in cyberspace of medical data from outerspace. Quantitative OCT data could be displayed with high reliability and enable collaborative communication of the digital medical data with the ground control on earth or promote an independent processing of data during long travels to save resources.

## Conclusions

The current study demonstrated that the OCT imagery can be jointly experienced and discussed among experts in a virtual reality cyber space in real-time overcoming spatial separateness and in a way they never could before. The csVR length measurements were, in average, smaller than the resolution limit of the underlying OCT recordings. Physical negative effects, such as nausea or motion sickness, have been eradicated to an acceptable level. To trust a VR application, a quantifiable and reproducible VR design was provided that extended to the cyberspace realm. The proposed csVR system allows access to medical data within seconds through direct integration with existing medical databases, such as the picture archiving and communication system (PACS) system^20^, which was developed according to the international standard DICOM 3.0. This enables faster and more reliable communication of medical data, so that not only experts^22^ but also lay people^17^ and even children^21^ can be exposed to this easily understandable csVR technology. A level has been reached where virtual digital medicine has become mature and valid^22^, not only locally in a clinical setting but also for data immersion worldwide in a flexible, rigorous, and protected environment.

## Methods

### Subjects

Three members from our study group simultaneously explored (Fig. 3) the utility of the proposed VR technology from geographically distant places (Lucerne, Switzerland, Barcelona, Spain, and London, UK).

### Hardware

All graders used HMDs, including two motion controllers (HP Reverb G2 VR Headsets Rev. 2, Hewlett-Packard Company, Palo Alto, United States). The VR system was tethered to gaming laptops (two Asus G14 GA401V, Microsoft Windows 10 home × 64, AMD Ryzen 9 4900HS with Radeon Graphics, 3.0 GHz, 8 cores, NVIDIA GeForce RTX 2060 with Max-Q design, 16 GB RAM, and one ASUS Zephyrus GX501GI, Windows 10 Pro × 64, Intel ® core i7-8750H 2.2 GHz 6 cores, 3 NVIDIA GeForce GTX 1080 Max-Q design, 2 GB RAM). The computers were connected to the internet using gigabit Ethernet and were communicating through a relay server. The relay server was running on a virtual host with six virtualized CPUs, each corresponding to an Intel Core CPU (Broadwell, IBRS) running at 2.2 GHz and 8 GB RAM.

### Software

The medical visualisation software Specto used in this project was created with the cross-platform Unity Engine (Unity Technologies, San Francisco, USA) and was extended with additional features for this work. The software supports loading any volumetric medical data from standard file formats, such as DICOM or Nearly Raw Raster Data (NRRD). The loaded data is then rendered via a custom ray-marching-based volume renderer, with no manual processing required. Multiple performance optimizations are used, such as empty space skipping^45,46^ and early ray termination, to achieve the necessary refresh rate of at least 90 Hz and thereby avoid motion sickness. During a collaborative session, several users can connect to a host and receive the loaded volumetric data via an encrypted transmission.

A connection to other users can be established directly via an Internet Protocol (IP) address or indirectly over a relay server. The relay server software provides an end-to-end encrypted room system and makes it possible for the VR host and VR clients to communicate with each other even through firewalls, which are common in a hospital network environment. The relay server is application independent and only forwards the underlying Transmission Control Protocol (TCP) communication. The VR client is free to initiate a separate Transport Layer Security (TLS) cryptographic protocol and negotiate with the VR host to choose the best encryption algorithm supported by both machines. In this architecture the VR applications can communicate through the relay server without running the risk of a compromised relay server getting access to sensitive medical data.

### Cyberspace virtual reality environment

The VR software was installed on each computer, which enabled the graders to take part in this study. After all graders had immersed themselves in cyberspace with their HMDs, they joined the VR arena after login into a password-secured environment. The arena consisted of a darkened room with four walls, a ceiling, and a floor. In the middle of the room, one volume-rendered OCT cube (4.5 mm × 3 mm × 1.9 mm) was made available for digital manipulation, which consisted of stacked 261 spectral-domain OCT cross-sectional images from a healthy macula from a right eye (Spectralis, Heidelberg Engineering, Heidelberg, Germany). The OCT scan angle was 30° with a scan pattern of 15° × 10°, interslice distance of 12 µm. Scan-enhanced depth imaging was activated, and each B-scan was averaged for 20 scans using the automatic averaging and tracking feature. This resulted in an image quality of 29 dB.

### Consensus and diameter measurements

Before grading, each grader received consensus documentation (Supplementary File Fig. S1). The graders then executed the tasks in a 5-h sequence, which included two 10-min pauses. The session started with an instruction by the most experienced user (PMM), who explained the VR room, the operation of the handles, and the safety measures to the others using seamless spatial-audio utility.

This was followed by consensus diameter measurements on different B-scans: A grader specified a diameter to be measured, which could be located within the retina but could also extend to the choroid. These two landmarks were initially depicted on a B-scan with the yellow marker tips of the handles, whereby the display was performed alternately by different graders. Preferably, the outer borders of vessels were used as the starting point or end point, respectively, as they displayed the highest contrast. The measurement line was visible only to the individual grader. The length of the measurement was also hidden on the measuring monitor. After the first line was measured, the remaining graders were engaged in measuring the identical line between the proposed landmarks. Finally, the values were unlocked on the measurement board with a click from the specific grader, and the values were discussed with each other. By masking the lines, the end points, and the measured values, maximum blinding of the measurements was ensured. The obtained values were locked from any changes and transferred to the host computer for storage as a Microsoft Excel file (Microsoft Corporation, Redmond, United States) for each single diameter with three measurements per identical diameter by clicking on an export button. All three measurements were then cleared to start a new run. This was repeated for each of the 10 consensus measurements. Afterwards, the same procedure was performed for the real-life-time diameter measurements until enough values were collected. All measured values were stored on the host computer and merged into one final file (Supplementary File S1) for further data analysis.

### Simulator Sickness Questionnaire and software evaluation

After exposure, the three participants were asked to complete a Simulator Sickness Questionnaire (SSQ)^47^ to assess nausea, disorientation, and oculomotor disturbances. For this purpose, a custom written script was applied, and the results were evaluated. Additionally, a software usability analysis was conducted to investigate the potential benefits and limits of the proposed application.

### Statistical analysis

The summary statistics mean, median, min, max, standard deviation, and coefficient of variation (CoV) were calculated for the recorded length measurements with the library pandas v1.5.2 in Python v3.10.9. For each of the 109 objects, the consensus length was defined as the mean of the three length measurements of graders g1, g2, and g3. The mean absolute differences between all pairs of graders and the consensus were calculated with the library NumPy v1.23.5^48^ yielding a distance matrix.

This distance matrix was used as input for metric multidimensional scaling (MDS). MDS converts distances among data points into positions of those data points in a two-dimensional coordinate system while preserving the distances as best as possible. MDS was performed using library scikit-learn v1.2.0^49^. The MDS plot was centered at the consensus.

All plots were all drawn with the library matplotlib v3.6.2^50^ using a red-green colourblindness-friendly colour-map^51^.

The influence of the independent variables “grader” and “line colour” on the dependent variable “measured length” was investigated with an analysis of variance (ANOVA). Since the size of the 109 measured objects varied from 0.23 mm to 3.97 mm, “object ID” was included as an additional independent variable. The effect of “object ID” is not of primary interest, but it was included as a covariate to adjust for when investigating the effect of “grader” and “line color.” Based on a quantile–quantile plot, four of the 327 data points were removed from the analysis because they violated the assumption of normally distributed residuals. A Type II sum of squares estimation was used. ANOVA was performed with the package car v3.0.2 using R Statistical Software v3.6.1^52^.

### Ethics declarations

Written informed consent was obtained from all graders. In addition, all study participants provided informed consent to publish the information/image(s) in an online open-access publication. All methods were carried out in accordance with relevant guidelines and regulations and all experimental protocols were approved by the local ethics committee (Ethikkommission Nordwest- und Zentralschweiz, ID EKN 2016-01948).

## Supplementary Information

Supplementary Video 1.

Supplementary Information 1.

Supplementary Information 2.

Supplementary Information 3.

## Data Availability

The source data underlying the graphs and charts presented in the main figures are available as Supplementary File S1. The first author should be contacted to request the data from this study.
