# Supplementary material for: Validation of collaborative cyberspace virtual reality oculometry enhanced with near real-time spatial audio
Source: Sci Rep. 2023 Jun 21;13:10076. doi: 10.1038/s41598-023-37267-x (PMC10284898; doi:10.1038/s41598-023-37267-x)
Supplement: Supplementary file 3 — Supplementary Information 2. [file 41598_2023_37267_MOESM3_ESM.docx]

[Supplementary File S2](https://arvo.silverchair-cdn.com/arvo/content_public/journal/tvst/937352/tvst-07-04-05_s02.docx?Expires=1677959418&Signature=V8L9Mi2mxtzDA-R8GL0cplJMFBZXi3NEl9Ka2w9-g18NP98TbuM5and71F1ruiMj~PVNhNm6Agrnb1Z-Xkyt-g186pe6Gliy2cq~bfMdZpZOoyf6Iu333WTzYZniCLmD-BRL9V6hTjfE53tjn7u14Gg-GneNY4IW6c-7Z2XxmlqrMS0eObePoRaVzqQrh0AkHqQDzCOOnhYqMhf2ZK-TedK334HPIh8rdTyKJzU1PFDe1U2EKNdl9~vlVpE53jSIhLAKh1qqwCxtL5cocLKJgNZTcrl03LPrV2Kd0~GpIowFW1w3iKX2myOQchwAI4zyejciKIQNoKde4oWERtujgA__&Key-Pair-Id=APKAIE5G5CRDK6RD3PGA). Summary of comments by the graders.

1. **The biggest strength of the csVR tool is**

Intuitive visualisation of 3D data for both clinicians and potentially patients.

The possibility of interaction with colleagues both with audio and video stream.

Ability to interact and explore the OCT image in real time, it was really easy to navigate in the csVR space. In particular, during the experiment I found that the VR tool is really helpful to analyse OCT images together with other doctors in remote locations in real time.

The ability to interact with other doctors and navigate in the OCT volume scan in real time.

The accurate audio communication was excellent, with a sense of spatial depth and the ability to acoustically localise the colleagues in the room.

Fast coupling to the VR handles without time, so that the measurements could be carried out virtually as in the physical world.

1. **The biggest limitation of the csVR tool is:**

The technical set up.

The reduced field of view of the VR glasses.

If the magnification was too large, a tremor became detectable.

1. **If I could improve one thing about the csVR tool, it would be:**

To include several options for image analysis:

• Real-time applications of segmentations

• Classification of lesions

• Set pinpoints on structures with labels

• Multiple-choice options for classroom teaching in cyberspace: E.g. which structure is this?
