# Supplementary material for: Validation of collaborative cyberspace virtual reality oculometry enhanced with near real-time spatial audio
Source: Sci Rep. 2023 Jun 21;13:10076. doi: 10.1038/s41598-023-37267-x (PMC10284898; doi:10.1038/s41598-023-37267-x)
Supplement: Supplementary file 4 — Supplementary Information 3. [file 41598_2023_37267_MOESM4_ESM.docx]

Title: Validation of collaborative cyberspace virtual reality oculometry enhanced with near real-time spatial audio

Authors:

Peter M. Maloca^*1,2,3^, Javier Zarranz-Ventura^4^, Philippe Valmaggia^1,2,3^, Balázs Faludi^5^, Marek Zelechowski^5^, Adnan Tufail^3^, Norbert Z. Zentai^5^, Hendrik P. N. Scholl^1,2^, Philippe C. Cattin^5^

Affiliations:

^1^Institute of Molecular and Clinical Ophthalmology Basel (IOB), Basel, 4031, Switzerland

^2^Department of Ophthalmology, University Hospital Basel, Basel, 4031, Switzerland

^3^Moorfields Eye Hospital NHS Foundation Trust, London, EC1V 2PD, UK

^4^Hospital Clínic of Barcelona, University of Barcelona, 08036, Spain

^5^Centre for Medical Image Analysis & Navigation, University of Basel, Allschwil-Basel, 4123, Switzerland

***Corresponding Author**

Peter M. Maloca

^1^Institute of Molecular and Clinical Ophthalmology Basel (IOB)

4031, Basel

Switzerland.

Email: peter.maloca@iob.ch

Tel: +41 61 265 92 14

**Supplementary File S1.** Summary of all measured cyberspace VR values.

**[Supplementary File S2](https://arvo.silverchair-cdn.com/arvo/content_public/journal/tvst/937352/tvst-07-04-05_s02.docx?Expires=1677959418&Signature=V8L9Mi2mxtzDA-R8GL0cplJMFBZXi3NEl9Ka2w9-g18NP98TbuM5and71F1ruiMj~PVNhNm6Agrnb1Z-Xkyt-g186pe6Gliy2cq~bfMdZpZOoyf6Iu333WTzYZniCLmD-BRL9V6hTjfE53tjn7u14Gg-GneNY4IW6c-7Z2XxmlqrMS0eObePoRaVzqQrh0AkHqQDzCOOnhYqMhf2ZK-TedK334HPIh8rdTyKJzU1PFDe1U2EKNdl9~vlVpE53jSIhLAKh1qqwCxtL5cocLKJgNZTcrl03LPrV2Kd0~GpIowFW1w3iKX2myOQchwAI4zyejciKIQNoKde4oWERtujgA__&Key-Pair-Id=APKAIE5G5CRDK6RD3PGA).** Summary of comments by the graders.

**Supplementary figure file S1. Summary of the proposed method and consensus for measuring cyberspace virtual reality diameter.** Each cyberspace grader was instructed by this document before grading. First, the cyberspace arena was introduced. The items of the arena include a rendered OCT volume and a single conventional cross-sectional image (as an example, the cross-sectional image number 188 is illustrated). The magnet symbol illustrates that in the current study, the cross-section OCT image was locked orthogonally to the volume and could be moved along the corresponding axis using the csVR handles.

In the video tutorial, a single OCT volume is manually magnified with the VR handles. It is then shown how to snap the cross-section plane onto the OCT volume by clicking on the magnet symbol. On the OCT cross-section image, two vessels are indicated by a grader. A diameter measurement is started by clicking on the "plus" sign on the result panel. The first measurement point is set on the cross-section image, again with a magnetic function built in so that the measurement point snaps securely within that specific measurement plane. The second measuring point is placed analogously on the second localisation. Both the measuring line and the measuring points as well as the measuring length are masked until the corresponding user clicks on the result panel to reveal them. In this example, the csVR diameter of one object was 0.824 mm. In the last picture, the corresponding results of all three graders for a specific diameter have been released for inspection.
